# Supplementary material for: The Pilot Study of Immunogenicity and Adverse Events of a COVID-19 Vaccine Regimen: Priming with Inactivated Whole SARS-CoV-2 Vaccine (CoronaVac) and Boosting with the Adenoviral Vector (ChAdOx1 nCoV-19) Vaccine
Source: Vaccines (Basel). 2022 Mar 30;10(4):536. doi: 10.3390/vaccines10040536 (PMC9028748; doi:10.3390/vaccines10040536)
Supplement: Supplementary file 1 [file vaccines-10-00536-s001.zip › vaccines-1557581-supplementary.pdf]

Supplementary Materials

1.Supplementary figure

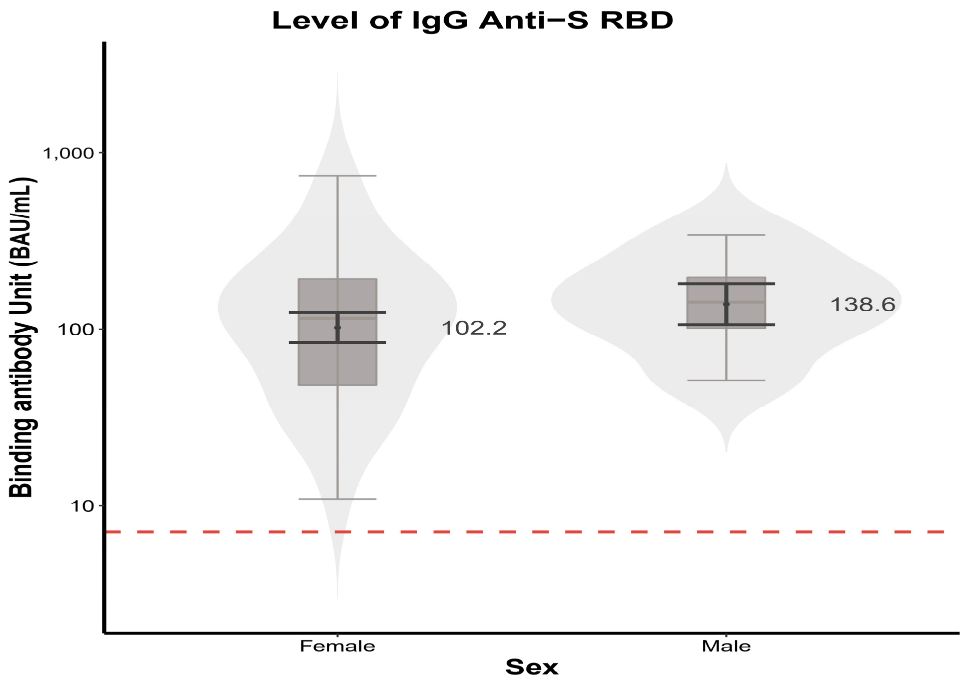

CoronaVac/CoronaVac

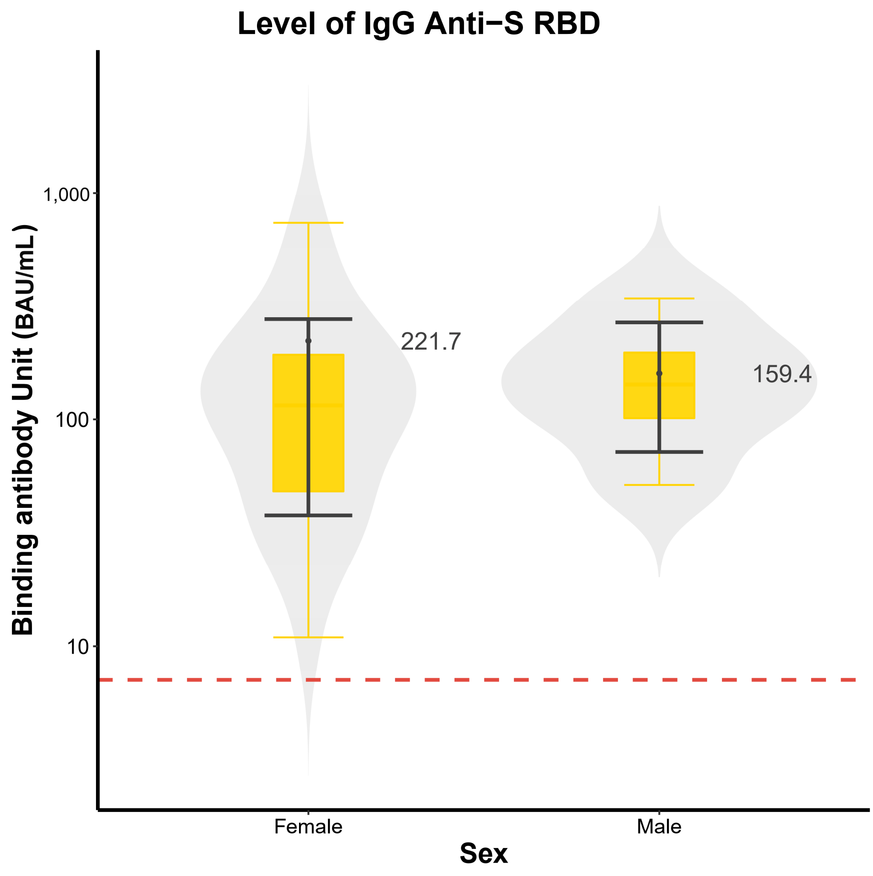

ChAdOx1/ChAdOx1

Supplementary Figure S1: Violin plots comparing level of IgG anti-SRBD titer between males and females in CoronaVac/CoronaVac group and ChAdOx1/ChAdOx1 group
Boxplots show geometric mean and IQRs. Serum was collected at 4 weeks.

In CoronaVac/CoronaVac group, The GMT of anti-SRBD titer was (138.6 BAU/mL, 95% CI 69.5 – 276.2) in male, (102.2 BAU/mL, 95% CI 68.3 – 152.7) in female.

In ChAdOx1/ChAdOx1 group, The GMT of anti-SRBD titer was (159.4 BAU/mL, 95% CI 58.1 – 437.2) in male, (221.7 BAU/mL, 95% CI 166.5 – 295.2) in female.

There was no statistically significant difference between males and females in either the homologous CoronaVac (p-value = 0.436) or homologous ChAdOx1 (p-value = 0.209) group.

2.Supplementary table

	CoronaVac/CoronaVac		ChAdOx1/ChAdOx1	
	Male	Female	Male	Female
N	6	26	7	40
geoMean	138.6	102.2	159.3	221.7
Mean	164.3	159.0	280.8	313.3
95% CI	69.5 – 276.2	68.3 – 152.7	58.1 – 437.2	166.5 – 295.2
p-value	0.436		0.209	

Supplementary Table S1: Comparison of the anti-SRBD levels between males and females in homologous CoronaVac and ChAdOx1 group
Additionally, we compared the anti-SRBD qualitative level (BAU/mL) between males and females using the Wilcoxon rank sum test on R software. There was no statistically significant difference between males and females in either the

homologous CoronaVac (p-value = 0.436) or homologous ChAdOx1 (p-value = 0.209) group.

Adverse events (AEs)	CoronaVac/ ChAdOx1	CoronaVac/ CoronaVac	ChAdOx1/ ChAdOx1	P-Value
	N=155	N=32	N=47	
**Total AEs (%)**	129 (83.26%)	18 (56%)	23 (49%)	0.02812
**Systemic**				
**Feeling Feverish (%)**	104 (67%)	1 (3%)	11 (23%)	<0.001
**Headache (%)**	51 (33%)	6 (19%)	5 (11%)	0.0057
**Myalgia (%)**	40 (25.81%)	2 (6%)	8 (17%)	0.0351
**Dyspnea (%)**	2 (1.29%)	0	0	1
**Paresthesia (%)**	1 (0.65%)	0	0	1
**Rash (%)**	3 (1.94%)	0	0	0.5357
**Fatigue (%)**	30 (19.35%)	2 (6%)	5 (11%)	0.098
**Diarrhea (%)**	4 (2.58%)	1 (3%)	2 (4%)	0.844
**Nausea And Vomiting (%)**	13 (8.39%)	4 (13%)	0	0.059
**Local**				
**Injected Site Pain (%)**	54 (35%)	8 (25%)	4 (9%)	0.0035

Supplementary Table S2: Comparison of Adverse Events in Each Vaccine Regimen after the second vaccination dose

The most common systemic AEs after the second vaccination dose within 4 weeks were feeling feverish (67.1%) in the CoronaVac- ChAdOx1 group and in the homologous ChAdOx1 group (23%) and headache (19%) in the homologous CoronaVac group

The most common local AE was injection site pain in all three groups (35.06% in the CoronaVac- ChAdOx1 group, 25% in the homologous CoronaVac group, and 9% the homologous ChAdOx1 group, respectively)
